# Lost Stone During Laparoscopic Cholecystectomy: Retrieval Using a Condom

**DOI:** 10.1155/1993/10187

**Published:** 1993

**Authors:** S. C. S. Chung, M. K. W. Li, A. K. C. Li

**Affiliations:** 1 Department of Surgery Prince of Wales Hospital The Chinese University of Hong Kong China; 2 Alice Ho Mui-Ling Nethersole Hospital Hong Kong China; 3 Department of Surgery The Chinese University of Hong Kong Shatin Hong Kong China

## Abstract

Laparoscopic cholecystectomy is becoming increasingly popular for the treatment of gall stone disease. In this technique, the gall bladder is dissected free under laparoscopic vision and then extracted. We report an interesting complication that occurred during extraction of a gall bladder containing a large stone and a novel method of overcoming the problem.


*HPB Surgery*, 1993, Vol. 7, pp. 67–68
Reprints available directly from the publisher
Photocopying permitted by license only

© 1993 Harwood Academic Publishers GmbH
Printed in the United States of America

### CASE REPORT

### LOST STONE DURING LAPAROSCOPIC CHOLECYSTECTOMY: RETRIEVAL USING A CONDOM

S.C.S. CHUNG, M.K.W. LI* and A.K.C. LI

*Department of Surgery, Prince of Wales Hospital, The Chinese University of Hong Kong and *Alice Ho Mui-Ling Nethersole Hospital, Hong Kong*

*(Received 11 March 1992)*

Laparoscopic cholecystectomy is becoming increasingly popular for the treatment of gall stone disease. In this technique, the gall bladder is dissected free under laparoscopic vision and then extracted. We report an interesting complication that occurred during extraction of a gall bladder containing a large stone and a novel method of overcoming the problem.

### CASE REPORT

A 41 year old man with a 4 cm diameter solitary stone in the gall bladder underwent laparoscopic cholecystectomy. The gall bladder was perforated during dissection of the gall bladder bed with the diathermy hook. The perforation was closed by application of a titanium clip across the perforation. The operation proceeded normally after the bile was aspirated from the Morrison's pouch and the area irrigated with saline containing tetracyline. The gall bladder was detached uneventfully from the liver. Despite enlarging the umbilical incision, the original tear in the gall bladder extended during attempted extraction of the gall bladder through the umbilicus. The 4 cm large solitary stone dropped out into the abdominal cavity. Grasping the stone at this stage with forceps was likely to result in fragmentation of the stone and contamination of the abdominal cavity. Retrieval of stone fragments would have been difficult or impossible. The other option was a laparotomy.

A condom was procured and inserted through the umbilical incision. Under laparoscopic vision, the stone was manipulated by forceps into the condom, which together with the stone, was then removed through the umbilicus. The patient made an uneventful recovery.

### *Discussion*

In laparoscopic cholecystectomy, extraction of the gall bladder containing large

Address correspondence to: Professor Arthur K.C. Li, Department of Surgery, The Chinese University of Hong Kong, Shatin, Hong Kong stones may be difficult. The stones may have to be fragmented or the umbilical incision enlarged before the gall bladder with stones can be removed. Perforation of the gall bladder is not uncommon in laparoscopic cholecystectomy. The operation can usually proceed normally after the perforation is closed by application of clips or Endoloops, (Ethicon, Somerville, New Jersey, USA). The patient usually suffers no ill effects provided the extravasated bile is sucked out and the peritoneal cavity generously lavaged with saline. Removal of gall bladder with a tear through the umbilicus requires special care as the tear may extend during manipulation and result in contamination of the abdominal cavity by bile and stones.

Once a large stone is free in the abdominal cavity, grasping it with forceps for retrieval runs the risk of fragmentation and further contamination. We have shown that its safe removal under such circumstances can be facilitated by manipulating it into a condom to avoid fragmentation before extraction. Specially designed bags for holding objects before laparoscopic removal may soon be on the market. Until they are available, the use of condoms in such situations helps to avoid contamination from stone fragmentation or a possible mini-laparotomy.

*(Accepted by S. Bengmark 15 March 1992)*

